# Analysis of lower limb prosthetic socket interface based on stress and motion measurements

**DOI:** 10.1177/09544119221110712

**Published:** 2022-07-12

**Authors:** Jinghua Tang, Liudi Jiang, Michael McGrath, Dan Bader, Piotr Laszczak, David Moser, Saeed Zahedi

**Affiliations:** 1 School of Engineering, Faculty of Engineering and Physical Sciences, University of Southampton, Southampton, UK; 2 Blatchford Products Ltd., Basingstoke, UK; 3 Skin Health Research Group, Faculty of Environmental and Life Sciences, University of Southampton, Southampton, UK

**Keywords:** Lower limb amputee, interface coupling, interface stresses, longitudinal, walking speed

## Abstract

The study was designed to establish a biomechanical assessment platform for the lower limb residuum/socket interface as a function of duration and speed of movement. The approach exploits an interface sensor which measures multi-directional stresses at the interface. The corresponding interface coupling motion was assessed using a 3D motion capture system. A longitudinal study, involving a trans-femoral amputee, was conducted with nine repeated level walking sessions over a 12-month period. The effect of walking speed on interface biomechanics was also assessed. Interface peak pressures and shear stresses in the range of 55–59 kPa and 12–19 kPa were measured, respectively, over all sessions in the 12 months study period at the posterior-proximal location of the residuum. The peak pressure and longitudinal shear values were found to fluctuate approximately 11% and 40% as against its maximum value, respectively, over 12 months. In addition, up to 12° of angular coupling and up to 28 mm of pistoning were recorded over a gait cycle, which was found to change by 29% and 45% respectively over the study period. The variation in walking speed, by altering self-selected cadence, resulted in changes of pressure and shear stresses at mid-stance of the gait cycle. In particular, as compared with self-selected cadence, for fast speed, peak pressure and peak longitudinal shear stress decreased by 5% and 33%, respectively. For slow speed, peak pressure and peak longitudinal shear stress increased by 7% and 17%, respectively. The corresponding angular and pistoning revealed a variation of up to 29% and 45%, respectively. This biomechanical assessment approach shows promise in the quantitative assessment of interface kinematics and kinetics for lower limb prosthetics, the usage of which could assist the clinical assessment of prosthetic socket fit.

## Graphical abstract

**Graphical Abstract:**
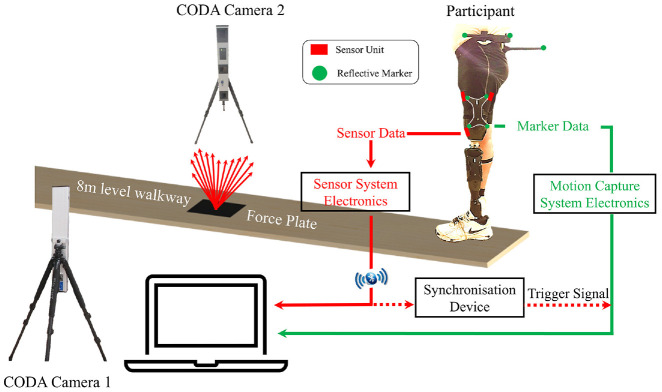

## Introduction

Since the inception of modular designs for lower limb prostheses in 1950,^1^ there has been a rapid surge of a range of advanced limb designs,^2^ the use of which has led to improved amputee care. However, many amputees still report issues related to socket fit and stump pain induced by prosthetic limbs, leading to poor satisfaction rate.^3^ This has become a challenge to UK National Health Service for lower limb amputee care.^4^

Similar to the gait cycle (GC) of able-bodied subjects, an amputee GC can be broadly divided into stance and swing phases. As the foot contacts the ground in stance phase, a ground reaction force is generated, acting upon the prosthetic foot. Subsequently, the load is transferred through the prosthetic components, for example ankle and knee of the trans-femoral amputees, to the socket interface.^5^ Load transfer from the ground to the socket can reach up to 270% of body weight and such load can occur up to 2 h/day. These multi-directional loads will be re-distributed over the residuum, via the socket. However, many amputees suffer from diabetes, vascular disease and/or peripheral neuropathy, thus the soft tissues covering their residuum are less tolerant to loading and highly susceptible to breakdown,^6^ leading to the formation of stump ulcers.^7^ Thus, the need to measure pressure and shear at the residuum/socket interface has been well recognised in this field.^3^

Indeed, residuum movement has been previously reported to be of magnitude of up to 40 mm in the axial direction^8^ and up to 7° in the sagittal plane.^9^ Excessive motions at the interface could also compromise gait stability and the effective safety of the amputee.^10^ It is thus important to evaluate the real-time interface biomechanics during a normal functional activity. We have developed such a comprehensive assessment platform combining the novel interface coupling model for kinematic measurements and a tri-axial pressure and shear sensing system for kinetic measurements.^11^

This longitudinal study has been designed to evaluate the characteristics of gait for a single participant on nine separate sessions over a 12 month period with an additional focus on the effect of walking speed on interface biomechanics.

## Materials and methods

### The participant

To ensure consistency and comparability across multiple sessions, a unilateral trans-femoral amputee (male, 29 years, body mass of 80 kg, height of 178 cm, post amputation of more than 20 years) participated in the study. He had a stable residual limb, free from infection and was able to walk without assistance. The participant was fitted with his habitual prosthesis throughout the study, including a supra-condylar suspension socket, KX06 knee and Echelon VT foot (Blatchford Products Ltd., Basingstoke, UK). A senior prosthetist verified the socket fit and the prosthetic alignment. The study was approved by the University Research Ethics Committee (ID: 12058 and ID: 6008) of the University of Southampton.

### The experiment setup and theory

**Figure 1.**
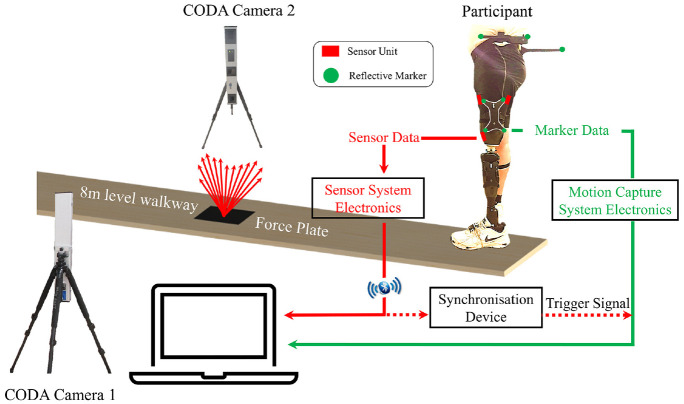
Schematic to depict the experimental test protocol in a gait laboratory, indicating the interface sensor and the marker placement on the participant, as well as the synchronisation between the sensor system and the 3D motion capture system.

Figure 1 shows the overall experimental setup, consisting of a tri-axial stress sensor system for the interface kinetic measurement and a conventional two-camera CODA 3D motion capture system (Charlwood Dynamics Ltd., Leicestershire, UK) to provide data for interface kinematics. CODA marker placement protocol has been followed according to CDL Gait-Cluster & Pelvic Slider version 1.06. On the prosthetic side, four reflective markers secured on a rigid cluster frame (outlined in white lines in Figure 1), were placed on top of the shorts, which was firmly strapped to the lower part of the socket that was not covered by the short. This was to ensure no relative movement between the reflective makers and the socket.

**Figure 2.**
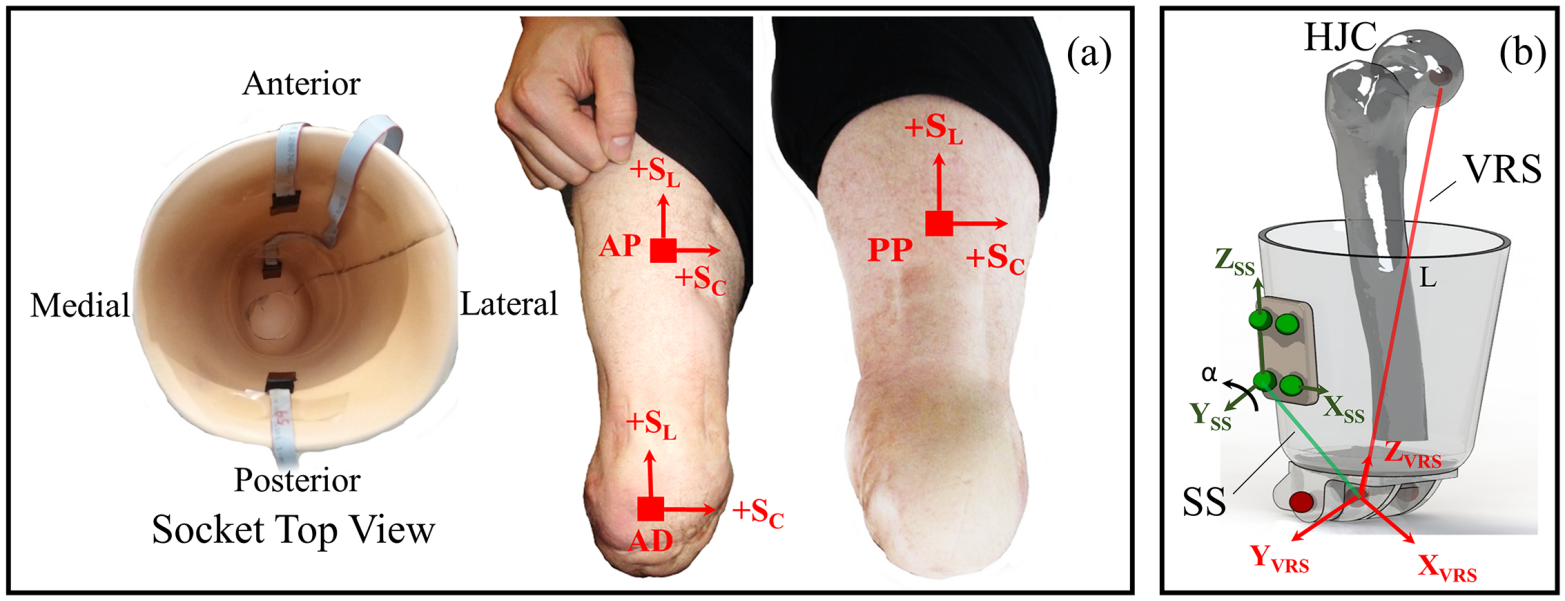
(a) Sensor placement on the inner socket wall and the corresponding locations on the residuum (b) an illustration to define virtual residuum segment (VRS), socket segment (SS), angular coupling (α) and pistoning (L).

The tri-axial pressure and shear sensor system are detailed in a previous paper by the authors.^12^ It consists of three sensor units and a wireless data acquisition unit (DAQ). Individual sensors were located at the anterior-proximal (AP), anterior-distal (AD) and posterior-proximal (PP) sites, providing a real-time output of interface pressure and shear during walking (Figure 2(a)). All sensor units are thin (1 mm thick) and very flexible. The suitability of its usage within the socket without causing any discomfort, nor the need to alter socket has been assured in our studies^11,12^ based on the amputee’s feedback and assessment of a senior prosthetist who were present throughout the tests.

The residuum/socket interface kinematics, involving pistoning and angular coupling, was based on marker data extracted from the 3D motion capture system, as detailed in our previous work.^13^ To review briefly, two separate segments are constructed at the socket interface, namely the Virtual Residuum Segment (VRS) and Socket Segment (SS), as shown in Figure 2(b). Local coordinate systems are defined for both the VRS and SS to calculate the relative angular movement between VRS and SS in real time. Angular coupling in sagittal plane (α) was chosen for analysis as it represents the plane with greatest movement based on the previous study.^13^ The dynamic pistoning (L), calculated as a dynamic displacement between the hip joint centre and prosthetic knee pivot centre, characterised the pistoning between the VRS and SS.

A synchronisation device was designed and implemented to allow simultaneous measurements of the interface stresses and couplings during a single walking test. Effectively, when acquisition of the marker position started, a 5 volt trigger pulse was transmitted from the motion capture system to register the start of the data acquisition.

### Walking tests

Physical makers for 3D motion capture and sensors for stress measurement were placed on the participant by the same investigator. The participant was subsequently asked to walk at self-selected speed along the 8 m level walkway with a force plate embedded approximately at its half-way point. At least eight clean trials were collected in a single evaluation session. A clean trial is defined as trials in which all markers are captured by the cameras and there was a complete single foot in contact with the force plate. In total, nine evaluation sessions were conducted over a 12 month period, out of which four were for assessing interface stress using the stress sensing system, four were for assessing interface coupling using the 3D motion capture system, and the final one was designed to combine both assessments simultaneously. For the first eight sessions, the stress and coupling measurement sessions were alternated with at least one month separation between the two consecutive sessions, over the 12 month period.

In the last session, the walking cadence, in steps per minute, was calculated based on the self-selected walking speed of the participant. The calculated walking cadence was then used to approximate slow and fast walking, represented by −20% and +20% of self-selected cadence. The participant was subsequently instructed to repeat the level walking tests at these slow and fast walking speeds, as aided by a metronome. A minimum of eight clean trials were conducted for both slow and fast walking tests.

The initial stump shape and mass distribution was assessed by a senior prosthetist. At the end of each session, body mass was recorded as an indication of any possible change in stump mass distribution, over the 12 month study period.

### Data processing and participant feedback

During each session over the 12 months, the peak values for each of the kinematic and/or kinetic measurements were extracted from each clean trial. Median values of measurement parameters, such as peak pressure and peak shear stress, were obtained from each session and used for analysis. In particular, the fluctuation of the medians, over the study period, were reported as percentages of the observed maximum out of all trials. In addition, the percentage change in peak interface kinematics and kinetics, associated with fast and slow walking speeds, were estimated with respect to those evident during level walking.

After each session, participant feedback was recorded on the health state of the residuum, change in socket fit due to body mass fluctuation and satisfaction level of the prosthetic limb.

## Results

### Longitudinal interface kinetic assessment over 12 months

**Figure 3.**
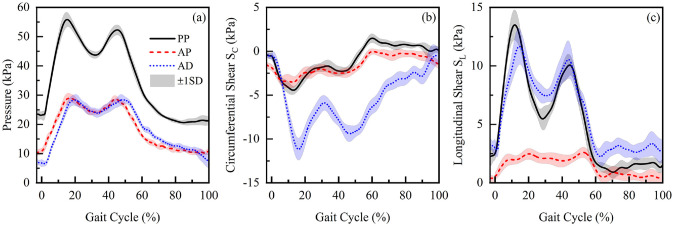
Mean and SD of (a) pressure, (b) circumferential shear stress (S_C_) and (c) longitudinal shear stress (S_L_) obtained at PP, AD, and AP locations in Session 2.

Figure 3 illustrates the pressure and the shear stresses in the circumferential (S_c_) and longitudinal (S_L_) directions measured at the three residuum locations, taken from a single measurement session. Pressure profiles reveal a double-hump profile during the stance phase at each location (Figure 3(a)). Peak pressures of up to 55, 30 and 30 kPa were recorded, in that particular session at PP, AP and AD locations, respectively. In the swing phase, the pressure was restored to the original value seen at initial contact (IC), that is, 0% GC. With respect to Sc, values of up to −5 kPa were measured at the two proximal locations with a corresponding value of up to −11 kPa measured at AD (Figure 3(b)). Up to 13 and 12 kPa of S_L_ were measured at PP and AD locations, respectively, with values of < 2 kPa at AP (Figure 3(c)).

**Figure 4.**
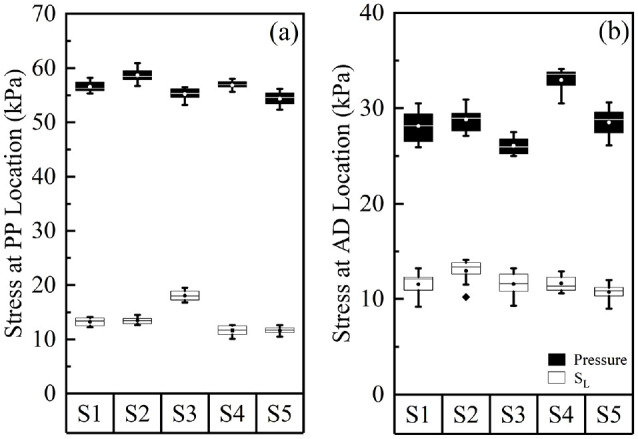
Peak pressure and longitudinal stress, S_L_, at (a) PP location and (b) AD location of the residuum, over five measurement sessions. In each session, results were calculated from 10 walking tests. The boxes are bounded by inter quartile range (IQR) and divided by a line, representing median value. Circles inside the box represent the mean value. Solid diamond symbol represents the outlier.

It is evident that the peak values for pressure and both shear stresses are higher for PP and AD locations when compared to the AP location. Figure 4 illustrates the median values for peak pressure and longitudinal stress across the five measurement sessions at the PP and AD locations. Over the 12 month period, there were up to 11% and 40% change in the median values of the peak PP pressure and S_L_, respectively. The corresponding changes at the AD location were 24% and 23%, respectively. The mean peak P and S_L_ values in Figure 4 showed no clear chronological trend, from Session 1 to Session 5.

### Longitudinal interface kinematic assessment over 12 months

**Figure 5.**
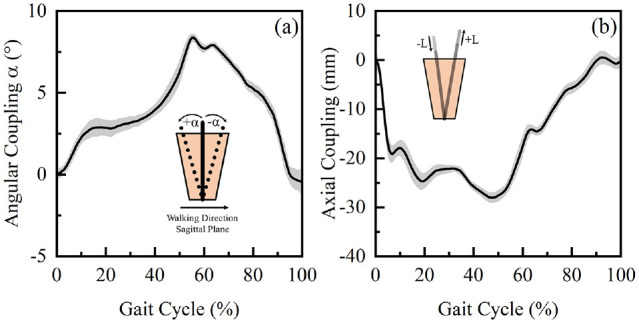
(a) Mean and SD of the interface angular coupling in sagittal plane, α and (b) pistoning, L obtained over a GC in Session 3.

Results indicated that the majority of the lower limb movement (α < 12°) is in the sagittal plane during the gait cycle (Figure 5(a)) with correspondingly small values in both coronal and transverse planes. In the stance phase, there was a general increase of α from IC to toe-off (TO), whereas in the swing phase, a decrease in α was evident such that it was restored to the original value at IC. Up to 29 mm of pistoning was measured over a GC (Figure 5(b)). There was a general decrease of L from IC to approximately 20% of the GC, suggesting distal movement of the residuum. Beyond this point, there was a slight increase of L until mid-stance phase. A subsequent decrease of L was evident between 30% and 50% of GC. During the remainder of the GC, L increases and is restored to the original value at IC.

**Figure 6.**
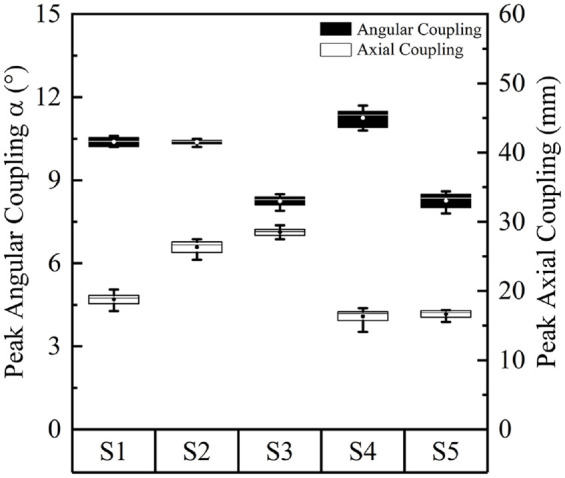
Absolute values of peak angular coupling in sagittal plane and pistoning, over five measurement sessions. In each session, results were calculated from eight walking trials. The boxes are bounded by inter quartile range (IQR) and divided by a line, representing median value. Circles inside the box represents the mean value.

Figure 6 illustrates the median values for peak α and L across the five measurement sessions. These results reveal changes in the kinematic parameters of up to 29% and 45%, respectively, over the 12 month period.

Approximately ±2% of body mass variation has been recorded over the 12 month study period. The senior prosthetist confirmed that the body mass variation measured over the study period is insignificant to result in a notable change in stump volume or shape.

### Effect of walking speed on interface biomechanics

**Figure 7.**
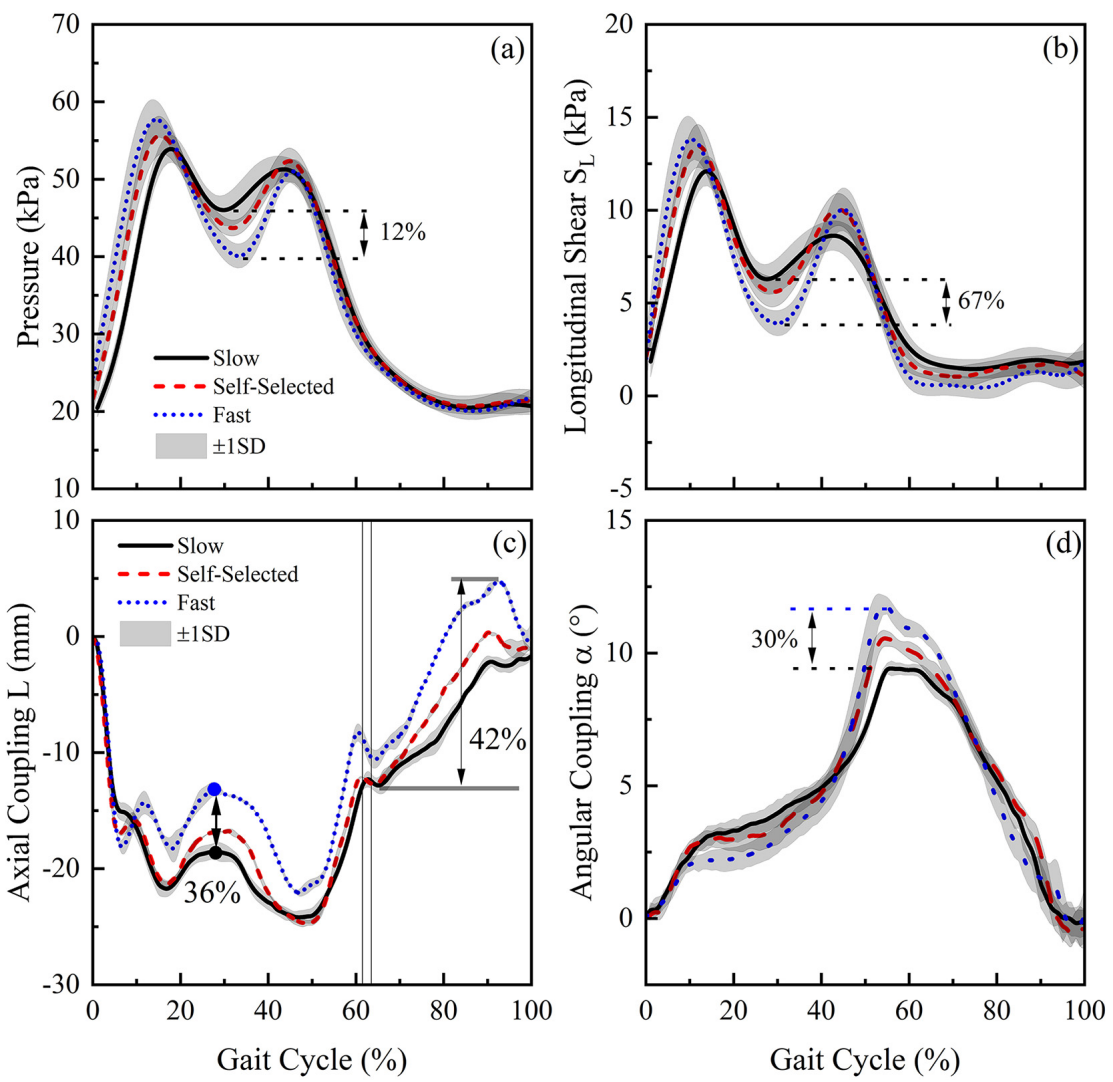
(a) Pressure and (b) longitudinal shear, S_L_ at PP location, obtained at slow, self-selected and fast walking speed, over a GC. (c) Pistoning, L and (d) angular coupling in sagittal plane, α obtained, obtained at slow, self-selected and fast walking speed, over a GC.

In the mid-stance trough, a pressure of up to 43 kPa was measured at the PP location at the self-selected walking speed (Figure 7(a)). The change in walking speeds resulted in a corresponding change of up to 12%, with respect to the values obtained in self-selected condition. In addition, S_L_ values of up to 6 kPa were obtained at PP location at self-selected walking speed in the mid-stance phase (Figure 7(b)). The changes in walking speeds resulted in a corresponding change of up to 67%.

With respect to L, values of up to −17 mm were estimated when walking at self-selected walking speed in the mid-stance (Figure 7(c)). The changes in walking speed resulted in a corresponding change of approximately 36%. In swing phase, up to 12 mm of residuum motion was measured at self-selected speed. The changes in walking speed resulted in a corresponding change of up to 42%. With respect to α, peak values of up to 10° were estimated when walking at the self-selected walking speed (Figure 7(d)).

## Discussion

This study investigates the change in residuum/socket interface kinematics and kinetics, over a period of 12 months and at different walking speed, using the techniques report previously.^11–13^ Interface stresses and coupling profiles were repeatedly measured over this time. Findings revealed that the magnitude of pistoning and angular couplings were similar to those in the seminal study by Convery and Murray.^9^

### Residuum/socket interface biomechanics over 12 months

Stresses obtained at PP and AD locations were chosen to analyse the fluctuation over the 12 month period, as they represent the key load bearing locations.^14,15^ The relatively low interface stresses at the AP location as compared with PP and AD is also evidenced in Figure 3. In general, we observed up to 11% (pressure), and 40% (S_L_) changes for the interface stresses, as well as 29% (α) and 45% (L) changes for the relative motion in the five measurement sessions over the 12 month study. The percentage values represent the absolute change in stress and pistoning of only 8 kPa and 12 mm, respectively, so any conclusions must be treated cautiously. Indeed, the authors propose further investigations of this type to evaluate the clinical significance of these findings.

Over the course of the study, there was no significant change in participant’s body mass and the mature stump was considered to be in a healthy condition. In addition, the participant used the same prothesis in each session with alignment checked by a single certified prosthetist and each measurement session was started at a similar time of day. In addition, feedback was recorded after each measurement session and neither discomfort nor change in socket fit were reported. The change in pressure and shear stresses observed in this study can be a direct result of several factors. These include the effect of donning and doffing of the prothesis which may influence the forces exerted on the residuum tissues, causing subsequent change in peak values of stress. In addition, residuum volume fluctuation^16^ may result in a change in socket fit condition leading to a re-distribution of stresses over the residuum. From an experimental perspective, the placement of each sensor may not have been reproducible, although effort was made to mark the sensor location on the inner socket wall as a guidance for sensor placement.

To the best of our knowledge, there is few reported studies focussing on interface stress variation over time, especially for that of trans-femoral amputees. Sanders et al.^17^ reported 30 kPa pressure variation (equivalent to 36% of mean peak pressure) and 5 kPa shear variation (equivalent to 43% of mean peak shear) over a 6-month period for trans-tibial amputees. We discuss here our trans-femoral amputee results alongside with this previously reported trans-tibial result with a view to providing preliminary comparison of longitudinal changes across these two main amputee populations. It is noted that all these reported changes for trans-tibial amputees^17^ are higher than the 11% measured at the PP location in this present study. This difference might be attributed to the bulk tissue mass on a trans-femoral residuum, which dominate the pressure distribution profile at proximal location of the residuum. By contrast, the change in pressure (approximately 24%) at the AD location in the present study was comparable to that previously reported. This could be predicted given that the present sensor was placed at the cut end of the femur, representing a bony prominence, and the pressures were dominated by the interaction between the bone and the socket, similar to the case on trans-tibial amputees.

In this study, change in S_L_ ranges in a range of 6%–23% was obtained ta AD location, which was lower than the value previously reported.^17^ This may be explained by the differences in the sockets in trans-tibial and trans-femoral designs. Indeed, trans-tibial sockets typically involve a tighter fit allowing more shear stress to be transmitted between the residuum and the socket.^18^

### Effect of walking speed on socket interface biomechanics

It was evident from Figure 7(a) that an increase in walking speed resulted in the decrease in pressure in the mid-region of the stance phase (approximately 30% of GC). This can be potentially explained by the increased movement of the body centre of mass in a vertical direction, associated with an increase in walking speed.^19^ The participant in the present study, who has been using the prothesis for over 10 years, has optimised his walking strategy to minimise the metabolic energy consumption at different walking speeds. Indeed, the increase in centre of mass movement in vertical direction is one of the strategies to conserve metabolic energy.^20^

The increase in walking speed resulted in a reduction in pistoning (Figure 7(d)) relative to the socket, at approximately 30% of GC. This reduction may be potentially explained by the efficient use of musculoskeletal work to maintain the residuum in a stable position and achieve foot-flat during mid-stance phase.

The increase in walking speed also resulted in an increased value of peak angular coupling in the sagittal plane. This may be predicted given the greater force at the PP location when compared to that at AP location, resulting in a pressure gradient as walking speed increases (Figure 3(a)). The pressure gradient will, in turn, translate the residuum movement towards the posterior location of the socket.

It is also worth noting that the variation of speed alone resulted in approximate changes of 12% and 67% in pressure and S_L_, with corresponding changes of 36% (pistoning) and 30% (angular coupling) in the relative motion at the interface. The changes, resulted from the variation in walking speed, were in similar ranges as compared with the changes observed over the 12 month period. This further implies that, although the interface kinetic and kinematic variations have been observed over a 12 month period, the degree of variation is equivalent to those induced by change in daily activities, for example, walking speeds. This was supported by the feedback from the participant during the various test sessions, namely, there was no change apparent over the study period. This may also imply that if quantified socket fit assessment is to be adapted in future clinics, we may require larger variations of interface biomechanics to evaluate socket fit levels. The combined kinetic-kinematic strategy adopted in the present study could represent a promising approach to assist such a quantitative assessment.

## Conclusions

In this study, a platform for the assessment of socket interface biomechanics was evaluated, using an interface stress sensor system (kinetics) and 3D motion capture system (kinematics). Each method was experimentally evaluated on a trans-femoral amputee, across five level walking sessions over a period of 12 months.

Preliminary results showed changes of up to 40% and 45% in interface kinetics and kinematics, respectively. In addition, the assessment platform was also found to be sensitive to changes in walking speed. Such a combined biomechanical assessment platform for the residuum/socket interface can potentially be used to aid the current socket fitting process and design of the patient-specific adjustable sockets and fully integrated limb systems, based on the socket movement and corresponding interface stresses.
